# Examining the Prevalence and Effects of Gender-based Violence in Academic Settings: A Systematic Review and Meta-analyses

**DOI:** 10.1177/15248380241289436

**Published:** 2024-10-23

**Authors:** Behdin Nowrouzi-Kia, Hong Yi Chan, Shangkai Zhu, Sharada Nandan, Ali Bani-Fatemi, Aaron Howe, Douglas P. Gross, Basem Gohar, Amin Yazdani, Vijay Kumar Chattu

**Affiliations:** 1 University of Toronto, ON, Canada; 2 University Health Network, Toronto, ON, Canada; 3 Laurentian University, Sudbury, ON, Canada; 4 Centre for Addiction and Mental Health, Toronto, ON, Canada; 5 The University of Hong Kong, China; 6 University of Alberta, Edmonton, Canada; 7 University of Guelph, ON, Canada; 8 Conestoga College, ON, Canada; 9 Datta Meghe Institute of Medical Sciences (DMIMS), Wardha, India; 10 Saveetha University, Chennai, Tamil Nadu, India

**Keywords:** gender-based violence, university, workplace violence, systematic review, meta-analysis

## Abstract

Gender-based violence (GBV) in the academic job sector is a critical issue that intersects with broader systemic and structural inequities, but research is limited. To study the prevalence, effects, and prevention measures of interpersonal GBV within the academic job sector, a meta-analysis and systematic review was conducted in accordance with the Preferred Reporting Items for Systematic Review and Meta-Analysis protocol. Rigorous searches were conducted across the databases PubMed, OVID, Scopus, Web of Science, and CINAHL, using specific keywords related to GBV, workplace, and virtual work environments, identifying papers published between January 2013 and February 2023. Studies were evaluated based on the Population, Intervention, Comparison, Outcomes framework. Data from papers were extracted and grouped by reported instances, and prevalence data for interpersonal GBV were reported in university settings, including in-person, hybrid, and virtual environments, and among men, women, and those who identify as 2SLGBTQ+. A random effects meta-analysis of proportions was conducted to evaluate the reported point prevalence rates of interpersonal GBV in academia between 2012 and 2015. Subgroup analyses were performed for university staff only, females only, and males only. Out of the 1,290 records, 16 studies met the inclusion criteria. The types of violence identified include sexual harassment, workplace bullying and online harassment, which affects career advancement, and employee well-being. The meta-analyses, conducted with a 95% confidence interval [CI], identified that 51.4% (95% CI [39.9%, 63.0%]) of university staff members experience GBV, with females, 59.3% [38.1%, 80.5%], experiencing greater rates than males, 44% [28.1%, 44.1%]. The findings underscore the need for institutional interventions to address interpersonal GBV in academic workplaces.

## Introduction

Gender-based violence (GBV) is a violation of human rights in which individuals face violence because of their gender, gender expression, gender identity, and/or perceived gender (Government of Canada, 2022). Interpersonal GBV in the workplace can manifest in various ways. Some examples include sexual violence, harassment, verbal mistreatment, bullying, coercion, psychological intimidation, inequality, and stalking, which is not only limited to physical violence but also encompasses verbal actions such as words, attempt to degrade, control, humiliate, intimidate, coerce, deprive, threaten, or harm another person (Government of Canada, 2022). These verbal actions are considered subtle or “yellow zone” incidents as the actions are often undetected or misunderstood (Banner et al., 2022). “Yellow zone” behaviors also include one-time events such as sexist comments and bullying (Richards & Rennison, 2022). Since these behaviors are subtle and do not raise to a level of policy violations, they are still harmful behaviors that contribute to unsafe work environments that negatively impact an individual’s experience at work (Banner et al., 2022; Richards & Rennison, 2022).

GBV can take many forms such as cyber, sexual, societal, psychological, emotional, and economic (Government of Canada, 2022). GBV has far-reaching consequences, as individuals’ experiencing sexual violence due to gender are more susceptible to developing concurrent mental and chronic health issues while also facing challenges in their work, compensation, daily activities, and caregiving responsibilities (World Health Organization, 2012). Moreover, victims of domestic violence experience both immediate and enduring psychological, behavioral, tangible, and mental consequences. By way of example, intimate partner violence incurs substantial economic and social burdens on women, their families, and societies, which may include instances of homicide or suicide, bodily injuries, unwanted pregnancies, abortion, and reproductive issues.

Banner et al. (2022) conducted a study in the United States, revealing alarming rates of harassment and inappropriate behavior within academic workplaces (Banner et al., 2022). Forty-three percent of participants reported experiencing workplace incidents of harassment. Specifically, the authors emphasized the occurrence of “yellow zone” incidents, which they define as subtle forms of sexual harassment, such as inappropriate jokes, bullying, derogatory comments, and hostile emails. Banner et al. (2022) found that 42% of participants reported enduring the types of incidents, underscoring the urgent need for institutional intervention to address the pervasive presence of such forms of harassment, particularly within the realm of “yellow zone” behaviors (Banner et al., 2022). In a previous investigation by Moutier et al. (2016) involving university faculty in New York, the study highlighted alarming rates of disruptive behaviors (Moutier et al., 2016). Notably, the research revealed that a concerning 29% of participants had encountered derogatory comments, with 25% experiencing anger outbursts and an equivalent percentage facing hostile communication. The study unveiled that 7% of respondents reported instances of sexual harassment.

These reports of interpersonal GBV go beyond the United States of America. For instance, a study conducted among Nigerian university women found that the prevalence of workplace incivility, bullying, and sexual harassment was 63.8%, 53.5%, and 40.5%, respectively (Agbaje et al., 2021). Additionally, a study among staff at a Swedish university discovered that 24.5% reported having to be exposed to sexual harassment (Agardh et al., 2022). These findings emphasize the need to cultivate a culture of respect in the learning environment as a crucial step in addressing and countering disruptive behavior and gender inequalities.

Gender inequality in the workplace is a multifaceted issue that not only results from GBV but also arises from institutional policies and practices that historically privilege men’s productivity and outputs over women’s (Linos et al., 2020). Structural GBV, characterized by systemic inequalities embedded in social, economic, and institutional frameworks, plays a critical role in perpetuating these disparities (Linos et al., 2020). Policies such as the lack of parental leave and insufficient support for women with children create significant barriers to women’s professional advancement, reinforcing a culture that undervalues their contributions and perpetuates gender inequality (Górska et al., 2021). In academia, opportunities for benevolent sexism are common as traditional, societal gender roles may provoke gendered pay gaps, unpaid labor roles assumed by women, misogynistic attitudes toward women’s work abilities, and limit upward mobility for women in leadership positions. For instance, Linos et al. (2020) highlights that many workplace policies fail to accommodate the needs of women, forcing them to choose between career advancement and personal responsibility. This structural inequity not only hinders women’s career progression but also creates an environment where interpersonal GBV, such as sexual harassment and bullying, can thrive.

Despite these alarming trends, limited research has addressed GBV in work environments within the academic sector, leading to an essential gap in our understanding of its prevalence, impact, and strategies for combating interpersonal GBV. This systematic review aims to fill this void by providing evidence-based insights, informing policy development, and guiding employers and employees on preventing, addressing, and rectifying GBV incidents in multiple work environments within the academic sector. Our study objectives were to examine the prevalence and impact of interpersonal GBV in physical, virtual, and hybrid work environments within the academic sector. We will identify the extent of interpersonal GBV among academics (students, facility, staff within post-secondary institutions) to evaluate the effectiveness of existing policies and interventions. We will also examine the consequences of interpersonal GBV on academics’ well-being and employment. By understanding the experiences of academics and the consequences of interpersonal GBV on employment, our study attempts to inform evidence-based strategies for creating safer and more inclusive work environments in academia.

## Methods

### Search Methods (Design and Strategy)

The present study was a part of a series of studies that looked at interpersonal GBV across occupational domains (i.e., healthcare, academia, and skilled trades), which are currently under review in peer-reviewed journals. To address our research inquiry concerning the impact of violence and harassment in virtual work environments within the academic sector, as well as the measures to prevent interpersonal GBV within the academic sector, a comprehensive and structured search was conducted following the guidelines outlined in the Preferred Reporting Items for Systematic Review and Meta-Analysis (PRISMA) framework (Page et al., 2021). This systematic review was registered with PROSPERO (CRD42023399684) before commencing. The search encompassed prominent academic databases, including PubMed, OVID, Scopus, Web of Science, and CINAHL, aiming to identify pertinent studies. Our search terms were designed to align with the research questions of the study, focusing on keywords such as “GBV,” “academic workplace,” “violence victim,” and “telework.”

The search strategy used specific keywords with Boolean operators, ensuring a full exploration of the selected databases. The search was conducted in May of 2023. Below is an exemplified representation of the search strategy executed in the PubMed database.

(“university”[Title/Abstract] OR “academia”[Title/Abstract] OR “college”[Title/Abstract] AND (“gender based violence”[Title/Abstract] OR “GBV”[Title/Abstract] OR “Harassing”[Title/Abstract] OR “Harassment”[Title/Abstract] OR “Cyberbullying”[Title/Abstract] OR “Cyberhate”[Title/Abstract] OR “Cyberharassment”[Title/Abstract] OR “gender discrimination”[Title/Abstract] OR “violence victim”[Title/Abstract] OR “exploitation”[Title/Abstract] OR “bullying”[Title/Abstract] OR “intimidation”[Title/Abstract]) AND ((“Virtual”[Title/Abstract] OR “web”[Title/Abstract] OR “Online”[Title/Abstract] OR “Remote”[Title/Abstract] OR “Digital”[Title/Abstract] OR “Internet”[Title/Abstract] OR “telecommuting”[Title/Abstract] OR “teleworking”[Title/Abstract]) AND (“work”[Title/Abstract] OR “employment”[Title/Abstract] OR “ labour “[Title/Abstract] OR “workplace”[Title/Abstract]))

The results from other databases are listed in Supplemental Appendix A.

### Selection Criteria and Eligibility

The study established its criteria for eligibility in advance, following the Population, Intervention, Comparison, Outcomes framework, and these criteria were evaluated and endorsed for content validity by the research team members (B.N.K., V.K.C., A.H., & A.B.). Intervention studies were included in the systematic review, but not in the meta-analytics procedures. There was no limitation based on the geography, type of study, or specific target population. Refer to Table 1 listed below for more information regarding the criteria we used for inclusion and exclusion.

**Table 1.** Inclusion Criteria and Exclusion Criteria.

Number	Inclusion Criteria
1	Studies that included documented instances or prevalence rates of diverse forms of interpersonal GBV, such as verbal abuse, sexual harassment, bullying, and similar manifestations, as well as GBV interventions in the workplace.
2	Any type of gender discrimination (any situation where a person is denied opportunities or misjudged because of their sex or gender identities).
3	In-person, hybrid or virtual work environment in a post-secondary institution (e.g., college or university).
4	All genders, including 2SLGBTQ+ community (two spirit, lesbian, gay, bisexual, transgender, queer, additional terminologies for sexual, and gender diverse communities).
5	Peer-reviewed journal articles published between January 2013 and February 2023 to reflect current and relevant information in the field.
6	Faculty, staff members, and/or students who were trainees employed at a post-secondary institution, that are of working age of (18–65 years old).
7	Articles reported in English language or has available English language translations.
	Exclusion Criteria
1	Non-peer-reviewed articles.
2	Articles published outside of the specific year range.
3	Unemployed participants.
4	GBV experienced outside of the work environment (i.e., domestic violence, spousal violence, intimate partner violence, violence by live-in partners).

### Study Selection

Following the guidelines of the Peer Review of Electronic Search Strategies statement (McGowan et al., 2016), we conducted the literature search. This process involved multiple independent searches performed by members of our interdisciplinary team (B.N.K., V.K.C., A.H., & A.B.). The reviewers took part in accurately recording and reporting data that was collected. The authors uploaded articles identified through the database search onto Covidence for compilation, organization, and evaluation purposes. Covidence also removed any duplicates found throughout the searches. All four reviewers evaluated the studies at the title and abstract phase, and full text phase to determine their eligibility based on the inclusion criteria. The review team met regularly to discuss any search strategy or methodological issues throughout the study selection and review process. Any disagreements at any phase of the study (e.g., title and abstract screening, searches and full-text screening) were addressed by the senior research members.

### Quality Assessment

Regarding quality assessment, four reviewers (B.N.K., V.K.C., A.H., & A.B.) conducted qualitative assessments utilizing diverse critical appraisal tools tailored to each study’s typology. *The Risk of Bias Instrument for Cross-sectional Surveys of Attitudes and Practices* by McMaster University (CLARITY Group, 2024) was employed for cross-sectional studies. Qualitative studies underwent critical appraisal using the checklist developed by the Center for Evidence-Based Medicine at the University of Oxford (Greenhalgh & Taylor, 1997). Additionally, *The Mixed Methods Appraisal Tool* was used to appraise mixed methods studies (Hong et al., 2018).

### Data Extraction

The synthesis and analysis of information encompassed study characteristics, prevalence of GBV, and relevant outcome findings. The authors recorded the following study characteristics: author name, year of publication, country, target population, and study design. To determine the prevalence rate of GBV in academia, two reviewers (S.Z., & S.N.) recorded the number of GBV-related events experienced by gender. GBV was determined based on the definition by the Government of Canada (2022), as well as any events related to violent and abusive experiences conducted in an academic setting. Prevalence had also been determined by the number of reported occurrences where respondents indicated gender being the cause. The prevalence rates of GBV were derived from divided the event (i.e., GBV_event_man) by totals (i.e., GBV_total_man). Data were recorded on a Microsoft Excel sheet for further analysis in R (R Core Team).

### Statistical Analysis

A random effects meta-analysis of proportions was conducted to evaluate the reported prevalence rates of GBV in academia from 2012 to 2015. These years were selected based on data availability from the included studies. The raw proportion was used to evaluate the variance of proportions because the proportions were mainly within 0.2 to 0.8. In terms of meta-analytic procedures, the underlying assumption is that the findings were independent. Two measures of heterogeneity were used to analysis study bias. The tau-squared represents the between-study heterogeneity, and it was estimated by the DerSimonian and Laird method. The *I*^2^ statistic was implemented as a measurement of the amount of between-study variance on the total observed heterogeneity. Subgroup analyses for variables of interest were performed, including analyses for males only, females only, and university employees only. A sensitivity analysis was conducted to assess the robustness of the synthesized results, which was reported using Funnel plots and Egger’s test for publication bias. All analyses, including sensitivity analyses, were conducted in R version 4.3.1 (MacOS; R Core Team). The “metafor” package (Version 4.4-0; R Core Team) was used for all meta-analytic procedures. Studies were excluded from the meta-analysis if they did not report adequate data to statistically compare GBV.

## Results

### Descriptive Findings

**Figure 1.**
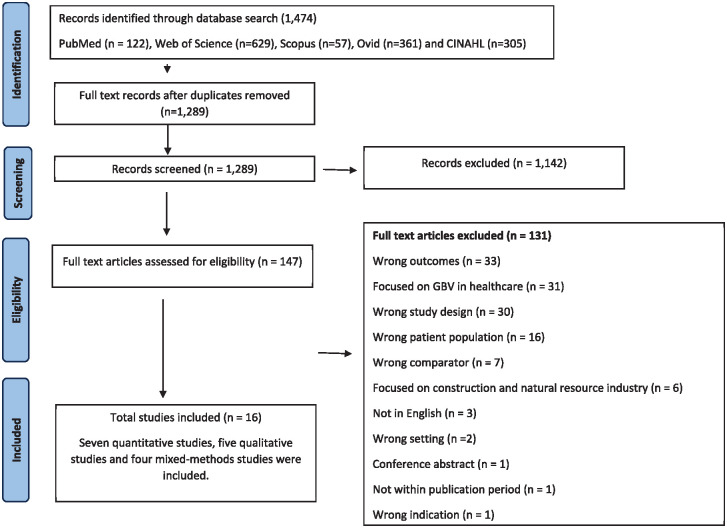
PRISMA flowchart.

A total of 1,290 records were identified after removing the duplicates. Following title and abstract screening, 1,142 papers were excluded as they did not meet the inclusion criteria, leaving 134 full texts to assess for eligibility. Ultimately, 16 articles were included in this review. Figure 1 displays the full screening process of the studies included.

Our review included a diverse range of studies from both the global South and global North covering various forms of workplace GBV in academia settings, including in-person and online/hybrid work environments. Of the total 16 reported studies on interpersonal GBV in academia, most of the studies are from United States (6, 38%), followed by Canada, France, Poland, Finland, Sweden, Senegal, Australia, South Africa, Zimbabwe, and Brazil with one each. There are seven cross-sectional studies, five qualitative studies, and four mixed methods studies included in this analysis of GBV in academia. There were seven studies that reported prevalence data and one intervention study; the remaining eight studies discussed instances of interpersonal GBV with academia, narratively. Study characteristics of the included studies are summarized in Table 2. Table 3 provides a summary of critical findings. Critical appraisal assessments for the cross-sectional studies are summarized in Supplemental Table 1 (refer to Supplemental Materials), qualitative studies are summarized in Supplemental Table 2 (refer to Supplemental Materials), and mixed methods studies are summarized in Supplemental Table 3 (refer to Supplemental Materials).

**Table 2.** Summary of the Included Studies as per the Eligibility Criteria (*n* = 16).

First Author	Year	Country	Target Population	Study Design	GBV Assessment
Academia					
Banner et al.*	(2022)	United States	University staff	Mixed Methods	Adapted #MeToo survey from existing literature; Online training module on perceptions and experiences with GBV.
Barr-walker et al.	(2021)	United States	University library staff	Mixed Methods	Survey on sexual and gender-based harassment perceptions + Institutional Betrayal and Support Questionnaire; Qualitative data: (a) Harassment experiences, (b) Disclosure factors, (c) Campus response, (d) Identity impact on harassment.
Berlingo et al.	(2019)	France	Postgraduate trainees in obstetrics and gynecology at University	Mixed Methods	Adapted survey from existing literature involving career aspirations, priorities, obstacles in academic career, comparison of obstacles based on gender; Qualitative data: Why women capable of academic careers hesitated to proceed with their career.
Conco et al.	(2021)	South Africa	University academic staff	Quantitative	A user-designed survey measuring workplace bullying asked academics if they had ever been bullied as faculty members: never, once, a few times, or often.
de Freitas Oleto and Palhares	(2022)	Brazil	Professors employed at University	Qualitative	User designed questionnaire containing open ended questions to measure heterosexist harassment at some point in their careers.
Górska et al.	(2021)	Poland	Academics working in a University	Qualitative	N/A
Gosse et al.	(2021)	Canada	Scholars and academics in University	Quantitative	Survey asked about factors that contributed to harassment, and events that led to harassment.
Lipton	(2021)	Australia	Academics working in a University	Qualitative	N/A
Martinez et al.	(2017)	United States	Academics working in University	Mixed Methods	Adapted survey from existing literature involving harassment and discrimination; Qualitative data: Open-ended prompts provided to assess motivation to leave former jobs in academia.
Mawere and Seroto	(2022)	Zimbabwe	Students working at University	Qualitative	Standardized interview guide (Hesse-Biber & Leavy, 2006; McMillan & Schumacher, 2014) was used to assess experiences of sexual harassments by lecturers to students and students to lecturers.
Moutier et al.	(2016)	United States	Health science faculty	Quantitative	User designed survey measuring workplace bullying. Faculty had been asked if they had experienced or observed three or more instances of inappropriate behavior or comments in their unit within the past year.
Muhonen	(2016)	Sweden	University teachers	Quantitative	User designed survey adapted from Ås (1978) to measure gender harassment. *“participants were asked if they had during the past 12 months experienced gender harassment at their work place in the form of: (1) encountering sexist or gender harassing language, (2) being made invisible e.g. in meetings, (3) being ridiculed, (4) being discriminated concerning their salary. Respondents rated the four items on a scale ranging from 1 (very rarely/never) to 5 (very often).”*
Oksanen et al.	(2022)	Finland	University Research and teaching staff	Quantitative	User designed survey measuring online harassment. Forms of hate include personal insults and violent threats, never to daily.
Sougou et al.	(2022)	Senegal	Health researchers at University	Qualitative	Descriptive, observational, cross-sectional qualitative interviews. Interview guide with open-ended questions was used on barriers relate to sex and gender.
Vargas et al.	(2020)	United States	University Medical school faculty	Quantitative	Sexual harassment measured using Sexual Experiences Questionnaire
Vargas et al.	(2021)	United States	University Medical school faculty and students	Quantitative	User designed survey measuring gender, heterosexist, and sex-based harassment.

*Note*. GBV = Gender-based violence.

* Study that Discussed Interventions for GBV.

**Table 3.** Summary Table of Critical Findings.

Systematic review	• Types of GBV reported by university staff members include sexual harassment, online harassment, gender discrimination, and gender harassment. • Workplace harassment had a negative impact on workers mental health and job productivity.
Meta-analyses	• Half of the universities’ staff experienced GBV while working at their respective workplace. • Female staff members reported experiencing GBV more often than males.

Cross-sectional studies had an overall rating of moderate-to-high risk of bias, with three ratings for moderate risk of bias and three ratings for high risk of bias. Qualitative studies had an overall rating of low-to-moderate risk of bias, with two ratings for low and two ratings for moderate. Finally, mixed methods studies were rated as having a low-to-moderate risk of bias. Any discrepancies between the two ratings were discussed among the research team to reach a consensus.

**Figure 2.**
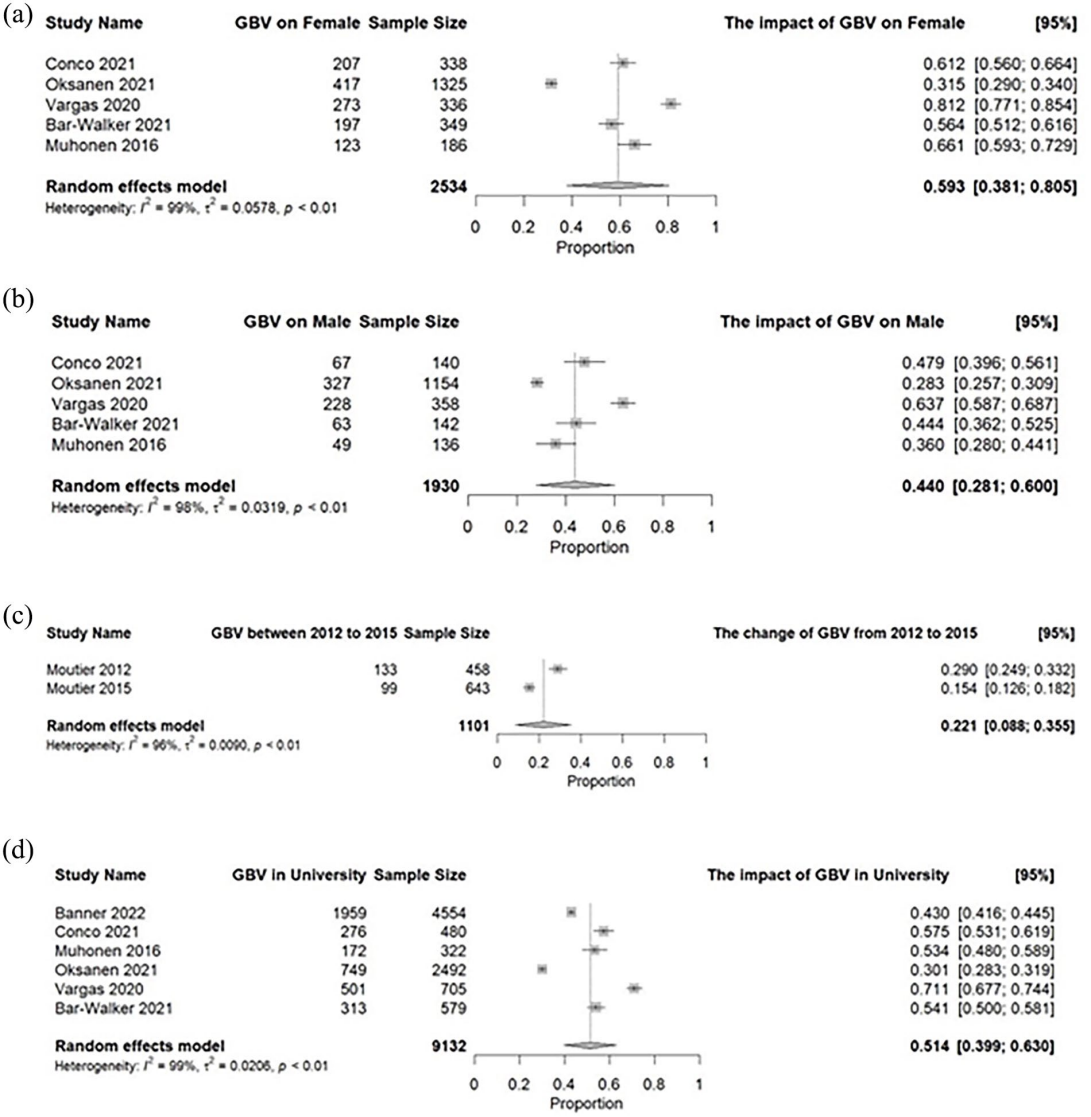
Gender-based violence meta-analytic results.(a) **Gender-based violence**, females: At least 30% of the female staff, faculty, and students/learners in the university experienced GBV, with the maximum reaching 81.2%. The pooled prevalence rate indicates that around 60% of female staff experienced GBV.(b) **Gender-based violence, males**: At least 30% of the male staff, faculty, and students/learners in the university experienced GBV, and the maximum could reach 63.7%. The pooled prevalence rate indicates that around 44% of male staff experienced GBV. (c) **Gender-based violence, 2012 to 2015:** In 2012, 29% of respondents in Moutier et al. study experienced GBV. The number decreased to 15.4% in 2015. The pooled prevalence rate is 22.1%, indicating that around 20% of respondents in Moutier et al. study experienced GBV.(d) **Overall gender-based violence, university:** At least 30% of the staff, faculty, and students/ learners within the university experienced GBV. In some studies, the prevalence rate could reach up to 70%. The pooled prevalence rate is 51.4% indicating more than half of the staff in the university experienced GBV. GBV, Gender-based violence.

Seven out of sixteen articles were chosen for the meta-analysis as they contained enough data to make statistical comparisons. Meta-analyses regarding the prevalence of GBV in academia can be found in Figure 2. The point prevalence rate of GBV among females was 59.3% (95% CI [0.381, 0.805]; *I*^2^ = 0.99). The point prevalence rate of GBV among males was 44.0% ([0.281, 0.600]; *I*^2^ = 0.98). The point prevalence rate of GBV in universities was 51.4% ([0.399, 0.630]; *I*^2^ = 0.99). The point prevalence rate of GBV in 2012 and 2015 was 29% and 15.4%, respectively. The compared point prevalence rate of GBV between 2012 and 2015 was 22.1% ([0.088, 0.355]; *I*^2^ = 0.96). We were unable to calculate the prevalence rate for 2LGBTQ+ individuals as only one study reported data on GBV experiences.

Funnel plots reporting GBV on males from 2012 to 2015 were created in Supplementary Figure 3. Egger’s test for publication bias was not significant for GBV in males (*p* > .3), females (*p* > .1), and in university settings (*p* > .1). However, the asymmetric pattern shown in the funnel plots indicates potential selection bias.

### Effects of Interpersonal GBV

#### GBV Among Women

Based on a large sample of academics and residents in research, Vargas et al. (2021) found that women were much more likely than males to face gender policing harassment, which is a form of harassment characterized by unfavorable treatment due to one’s gender role from staff. In addition, female members of underrepresented groups, including Asian, Asian Americans, or Pacific Islanders experienced greater rates of racialized sexual harassment committed by insiders (Vargas et al., 2021).

In their initial investigation of sexual harassment among California university library staff, Barr-Walker et al. (2021) discovered that 54% of workers had either personally experienced or seen sexual harassment at work (Barr-Walker et al., 2021). Additionally, according to Sougou et al.’s (2022) study of obstacles to professional advancement of women researchers in West Africa, women in Senegal found it more difficult to advance to leadership positions due to institutional policies, which deepened gender disparities and gender-insensitive organizational culture (Sougou et al., 2022). Martinez et al. (2017) reported that women in academia experienced higher gender discrimination and harassment than male counterparts (Martinez et al., 2017). Similarly, Muhonen (2016) observed in a Swedish study that gender harassment was more widespread among women university lecturers and researchers (Muhonen, 2016).

#### GBV Among Men

In a study conducted by Mawere and Seroto (2022) in Zimbabwe, the focus was on investigating the occurrence of harassment by learners who were women toward men teaching in academia (Mawere & Seroto, 2022). The study confirmed the existence of such harassment, highlighting the significance of fostering a culture of respect within universities. This culture would ensure that interactions between students and lecturers occur without the fear and occurrence of harassment.

Research from the United States examined the prevalence and consequences of sexual harassment in a university medical school, revealing a notable disparity in the likelihood of men experiencing GBV compared to women. Vargas et al. (2020) found that 82.5% of women and 65.1% of men reported at least one incident of sexual harassment from individuals within their workplace over the past year (Vargas et al., 2020). The study also revealed that workplace harassment had negative implications for mental health symptoms, job satisfaction, and feelings of safety, while also increasing turnover intentions among healthcare professionals.

Berlingo et al. (2019) investigated the relationship between gender and academic medicine job advancement (Berlingo et al., 2019). The study discovered that 40% of women and only 3% of men reported experiencing discrimination due to their gender among obstetrics and gynecology residents.

#### GBV Among 2SLGBTQ+

Vargas et al. (2021) found that individuals in the 2SLGBTQ+ community were at a higher risk of experiencing heterosexist harassment from both internal and external sources compared to cisgender heterosexual participants (Vargas et al., 2021). Heterosexist harassment in the workplace is defined as violent and insensitive verbal and symbolic behavior directed to non-heterosexual individuals in the workplace (De Freitas Oleto & Palhares, 2024). Moreover, de Freitas Oleto and Palhares (2024) investigated the experiences of Brazilian gay professor’s academics to heterosexist harassment (De Freitas Oleto & Palhares, 2024). The study revealed that instances of harassment were more overt when the professor exhibited more effeminate traits. No studies were identified that investigated GBV among individuals with diverse gender identities (i.e. the transgender and non-binary community).

#### Sexual Harassment and Bullying (Yellow Zone)

According to a study by Banner et al. (2022) in the United States, 43% of participants had experienced inappropriate behavior at work, and 42% reported having encountered a “yellow zone incident” (such as inappropriate jokes, derogatory remarks, or hostile emails) while employed by the university (Banner et al., 2022). The study also revealed that 28% of respondents had experienced sexually offensive remarks, 23% had experienced sexual topics being brought up that made them uncomfortable, and 19% had experienced comments about their body or appearance that were inappropriate. The authors concluded that there is a critical need for an institutional response to “yellow zone” kinds of harassment.

Moutier et al. (2016) discovered alarming rates of other disruptive behaviors among university faculty, including derogatory remarks (29%), angry outbursts (25%), and hostile communication (25%), as well as some levels of sexual harassment (7%) indicating the need to “improve a culture of respect in the learning environment” to address disruptive behavior (Moutier et al., 2016). Similarly, Conco et al. (2021) investigated bullying frequency and determinants among postgraduate students who are registrars/residents undergoing specialist training and discovered that 58% of respondents had experienced bullying, and 44% had experienced it more than once (Conco et al., 2021).

#### GBV in a Virtual Setting

According to a Canadian study by Gosse et al. (2021), factors like gender and physical appearance appear to contribute to online harassment (e.g. receiving negative comments online, zoom-bombing, doxing, etc.) that academic professors as well as graduate students experience (Gosse et al., 2021). It was suggested that universities expand their definition of workplace safety to include virtual environments. Another Finnish research study by Oksanen et al. (2022) reported that online abuse caused psychological discomfort and generalized mistrust among 30% of the respondents who belonged to minority groups (Oksanen et al., 2022). Derogatory remarks or inappropriate jokes, aggressive email, or verbal contact decreased job productivity for that person or others in the unit. Angry outbursts were just a few of the behavioral issues that have been seen or experienced.

### Interventions for GBV

Only one study reported interventions within the workplace to aid those experiencing GBV. A study by Banner et al. (2022) examined participant responses to an online training module aimed at advancing respectful and inclusive working environments, with a focus on reducing incidents of “yellow zone” sexual harassment and misconduct (Banner et al., 2022). The training module consists of three case studies involving potential sexual or gender harassment (Banner et al., 2022). Each case study includes options for how the employee should respond to each scenario such as reaching out to support the victim, become a bystander, file a report, or call 911 (Banner et al., 2022).

Fifty-four percent of participants who completed the training module reported that it provided a process for changing the culture on campus. Additionally, 70% of participants reported that it helped them understand what gender-based harassment is, with only 15% of participants reporting that they felt that the training did not adequately address gender-based harassment. Moreover, the study also highlights that there was still confusion over how to properly address and report “yellow zone” behaviors in practice, which calls for more robust and clear training modules to be developed.

## Discussion

The findings presented provide a comprehensive understanding of the prevalence of GBV within academic settings. GBV was reported in university settings, including in-person, hybrid, and virtual environments, and among men, women, and those who identify as 2SLGBTQ+. Seven of the included studies included in our meta-analysis were published between 2012 and 2015. These insights underscore the critical importance of adopting a multifaceted and intersectional approach to address GBV within academia effectively. In our subgroup analyses, we found a higher prevalence of GBV in females compared to males; however, the 95% confidence intervals were overlapping and wide indicating significant heterogeneity in the precision of the estimates for each study included. Furthermore, between study differences in reporting and measurement of instances of GBV, including small sample sizes of study participants, may be potential reasons for the variability in the pooled point prevalence calculated. Therefore, it is not conclusive whether there is a significant difference in interpersonal GBV experienced in academia between female and males.

Academia has continued to be a gendered organization with prominent hegemonic masculine characteristics (Jewkes et al., 2015). The workplace culture in academic continues to promote hypermasculinized behaviors, including competitiveness, stoicism, autocratic leadership, dominance, and a hierarchical access to power based on male (Fernando & Prasad, 2019; Täuber et al., 2022). Even though efforts have been made to promote gender equality and structural change, power dynamics continue to be unequally distributed with social discourses encouraging cultures of silencing, invalidation, and minimization or dismissal of reports of interpersonal GBV at an institutional level (Fernando & Prasad, 2019). Academic institutions are not only shaped by their formal structure, but also by the informal values they project, some of which contribute to the tolerance of sexual harassment and influence, and the way institutions respond to said harassment (Hearn et al., 2022).

The study by Banner et al. (2022) highlights a disturbingly common occurrence of harassment and inappropriate behaviors among academic professionals (Banner et al., 2022). Such experiences, ranging from workplace incidents to “yellow zone” occurrences, bring to the forefront the pressing need for academic institutions to take proactive measures in addressing and preventing GBV. Our findings resonate with those of Jagsi et al. (2023), who revealed alarming rates of sexual harassment, cyber incivility, and poor organizational climate within the academia, particularly among those belonging to minority groups such as women and those from underrepresented groups (e.g., LGBTQ+ status; Jagsi et al., 2023). As such, there is a dire need to cultivate a culture of respect and a sense of security by considering the effectiveness of internal anti-harassment policies within the academic environment. Currently, those who experience GBV are subject to internal scrutiny regarding the legitimacy of their reporting of GBV and conferral of partial responsibility for their victim status. We agree with the previous studies that have suggested there be an external complaint process to review and provide actionable recommendations to re-dress GBV in academic workspaces (Hershcovis et al., 2021; O’Connor et al., 2021; Täuber et al., 2022). This would protect GBV reporting channels by maintaining anonymity and promote appropriate justice for the accused and the victim. Review of anti-GBV and anti-harassment policies by persons from intersectional groups, those with lived experiences, and systematically reducing the involvement of those in hierarchical “power” positions.

The findings also illuminate gender-specific variations in the experiences of GBV. Those who identify as women, as demonstrated by Conco et al. (2021) and Berlingo et al. (2019), are disproportionately affected, facing increased odds of workplace bullying and experiencing identity-based harassment (Berlingo et al., 2019; Conco et al., 2021). Women are typically at a greater risk of experiencing workplace bullying due to being underrepresented in the workforce, particularly in higher position jobs (Araneda-Guirriman et al., 2023). Furthermore, the inherent characteristics of academia, in terms of academic work productivity and career progression, require conformist behavior, to be selected as a “good fit” for a limited number of faculty positions or promotions. This can lead to them feeling more inferior and vulnerable compared to their male counterparts as they may assume unpaid work assignments, have increased pressures to perform, and be forced into stereotypical roles within academia (e.g., support staff, teaching assistants, or non-advancing roles; Rosander et al., 2020). For instance, in an academic context, merely 36% of women make up senior university positions including full professors, deans, and university leaders (Bothwell et al., 2022). Moreover, 2SLGBTQ+ individuals are found to be particularly vulnerable to heterosexist harassment (HH), indicating the need for specialized interventions to ensure their safety and inclusion (Vargas et al., 2021). These findings echo the notion that GBV affects individuals in complex and interconnected ways, necessitating a holistic approach to its prevention and mitigation.

The implications of these findings in relation to research, practices, and policy are summarized in Supplemental Table 4 (see Supplemental Material). The observed prevalence of GBV within academic institutions has far-reaching consequences, affecting the well-being, mental health, and career trajectories of staff and students employed at the university. As noted in some studies, GBV has a negative impact on job satisfaction, mental health symptoms, and safety perceptions, underscoring the urgency of addressing GBV to create conducive academic environments (Conco et al., 2021; Moutier et al., 2016). For instance, a study conducted by Raj et al. (2020) found that individuals with an experience of sexual violence, specifically women, were more vulnerable to symptoms of depression and anxiety. These findings align with Gosse et al. (2021) and Górska et al. (2021), who emphasized the need to broaden workplace safety measures to encompass online environments and challenge gender inequalities within academia (Górska et al., 2021; Gosse et al., 2021).

The findings align with and contribute to a broader discourse on creating inclusive academic environments. The interconnectedness of GBV experiences across different gender identities necessitates an inclusive approach that recognizes and addresses the unique challenges faced by women, individuals from the 2SLGBTQ+ community, and other marginalized groups. Moreover, there have been several incidents across Canadian and American campuses where individuals belonging to the 2SLGBTQ+ community have experienced violent incidents due to their gender. In June of 2023, a violent attack occurred on the Waterloo campus where two students and a professor were injured during a gender studies course (Green, 2023). However, it is not just these severe incidents that are of concern. Less severe forms of violence and harassment, such as benevolent sexism, microaggressions, derogatory comments, or subtle discrimination, also contribute to a prejudicial academic environment. Microaggressions are often unintentional biases that can negatively impact individuals over time. For instance, a woman faculty member in science shared a frequent comment from a male colleague humorously questioning if her recent promotion could be a threat to her husband’s masculinity. These subtle biases can undermine the faculty members’ progress (Haynes-Baratz et al., 2022). The rise of hate-motivated incidents directed at individuals as a part of the 2SLGBTQ+ community highlights an urgent need to develop policies and interventions that protect and support all gender identities on campus. As Berlingo et al. (2019) and Górska et al. (2021) suggest, fostering a culture of respect and dismantling gender-based discrimination are pivotal steps in creating academic environments that promote growth and innovation, reinforcing the urgency of addressing GBV within academia (Berlingo et al., 2019; Górska et al., 2021). The need to address identity-based harassment, enhance workplace safety in online environments, and challenge gender disparities resonates across studies (Conco et al., 2021; Górska et al., 2021; Gosse et al., 2021).

### Recommendations

Hierarchical structures exist within academic workplaces where senior staff have immense power and influence over junior staff and faculty, creating a power imbalance. In fact, many junior staff rely on senior staff for career advancement (Fernando & Prasad, 2019; O’Connor et al., 2021; Täuber et al., 2022). Often, with these power imbalances in place, it creates an environment difficult for people in low-ranking positions to come forth with their experiences of GBV due to being seen as “trouble” or jeopardizing their opportunity to advance their career (Fernando & Prasad, 2019). This further exacerbates the problem when most higher position staff are male dominated (O’Connor et al., 2021; Täuber et al., 2022). With power imbalances apparent in academia, there is a strong need for interventions that tackle this issue to protect and support potential victims. Canada being the first to create the *Psychological Health and Safety in the Workplace Standard* serves as a guideline for providing 13 recommendations on mental health (Mental Health Commission of Canada, 2024). By way of example, psychological and social support is an essential recommendation as it entails fostering a secure space where individuals may voice their worries and ask for assistance without fear of reprisal. This component deals with the requirement for systems that offer emotional and social assistance to victims of assault and GBV. It also encourages organizational procedures that provide equal weight to each worker's mental health, irrespective of hierarchy. Moreover, the Temerty Faculty of Medicine at the University of Toronto currently has a system, referred to as *Learner Mistreatment*, that allows medical and rehabilitation science students to report incidents of harassment or other forms of workplace violence anonymously (University of Toronto, 2024). They have designated leaders from intersectional groups and medical discipline to review mistreatment incidents. This tool also ensures confidentiality to protect the identities of those reporting and encourage those to speak out against interpersonal GBV incidents in the workforce. Further evaluation of these tools is required to determine whether they effectively address and re-dress incidents of GBV or merely increase reporting channels with no justice for the victim.

## Limitations

There are a few limitations to consider when interpreting the findings of this study. The studies reviewed may be subject to publication bias. This threatens the accuracy of examining the prevalence of GBV across academic settings. To address this concern, we reviewed gray literature and dissertations in addition to reviewing published, peer-reviewed studies. We were also unable to evaluate data reported from collages as the studies used in the review all came from university populations, which potentially reduces the generalizability of our findings. Data used for the meta-analysis were taken from cross-sectional and mixed methods studies, which relied heavily on self-reported measures, and were only published in English. Moreover, we were unable to find articles discussing interventions of GBV, so we cannot provide recommendations on how to mitigate the effects and occurrence of GBV in the workplace. Considering the study’s design limitations, we cannot confirm that the rates identified directly relate to GBV in academic settings. However, results show how GBV is still prevalent in many academic institutions.

## Conclusion

This systematic review and meta-analyses study on GBV within academia, focusing on the unique experiences of men, women, and 2SLGBTQ+ individuals, fills a significant gap in existing research. The findings underscore the urgent need for institutions to address various forms of harassment, from verbal to online, and cultivate a culture of respect and equality in academic settings. The impact of GBV on mental health, job satisfaction, and career progression calls for targeted interventions and robust policies. By recognizing and understanding the differential experiences of individuals in academia, this study paves the way for more inclusive and safer academic environments.

In conclusion, the findings discussed emphasize the pressing need for a concerted effort to address GBV within academia. The experiences of men, women, and 2SLGBTQ+ individuals underscore the complexity of this issue and the necessity of a multifaceted and intersectional approach. While these studies offer valuable insights, they also signal the need for further research to deepen our understanding of the dynamics of GBV within academia and to evaluate the effectiveness of interventions across different academic settings and cultural contexts. Only one study evaluated the effects of a GBV training module on improving participants’ knowledge on sexual and gender-based harassment (Banner et al., 2022). Future research should focus on evaluating whether training modules effectively reduce GBV within academia in terms of reducing reporting rates and promoting safer working environments. Likewise, most studies were pooled from the USA, highlighting the need to expand this investigation across other geographical and cultural contexts. Future research should expand their samples to include more 2SLGBTQ+ participants. Ultimately, by collectively advocating for change, academia can work toward safer, more respectful, and inclusive environments for all individuals.

## Supplemental Material

sj-docx-1-tva-10.1177_15248380241289436 – Supplemental material for Examining the Prevalence and Effects of Gender-based Violence in Academic Settings: A Systematic Review and Meta-analyses Supplemental material, sj-docx-1-tva-10.1177_15248380241289436 for Examining the Prevalence and Effects of Gender-based Violence in Academic Settings: A Systematic Review and Meta-analyses by Behdin Nowrouzi-Kia, Hong Yi Chan, Shangkai Zhu, Sharada Nandan, Ali Bani-Fatemi, Aaron Howe, Douglas P. Gross, Basem Gohar, Amin Yazdani and Vijay Kumar Chattu in Trauma, Violence, & Abuse

sj-docx-2-tva-10.1177_15248380241289436 – Supplemental material for Examining the Prevalence and Effects of Gender-based Violence in Academic Settings: A Systematic Review and Meta-analyses Supplemental material, sj-docx-2-tva-10.1177_15248380241289436 for Examining the Prevalence and Effects of Gender-based Violence in Academic Settings: A Systematic Review and Meta-analyses by Behdin Nowrouzi-Kia, Hong Yi Chan, Shangkai Zhu, Sharada Nandan, Ali Bani-Fatemi, Aaron Howe, Douglas P. Gross, Basem Gohar, Amin Yazdani and Vijay Kumar Chattu in Trauma, Violence, & Abuse
