# Supplementary material for: Examining the Prevalence and Effects of Gender-based Violence in Academic Settings: A Systematic Review and Meta-analyses
Source: Trauma Violence Abuse. 2024 Oct 23;26(4):755–68. doi: 10.1177/15248380241289436 (PMC12397552; doi:10.1177/15248380241289436)
Supplement: sj-docx-1-tva-10.1177_15248380241289436 – Supplemental material for Examining the Prevalence and Effects of Gender-based Violence in Academic Settings: A Systematic Review and Meta-analyses [file sj-docx-1-tva-10.1177_15248380241289436.docx]

**Supplementary Table 1:** Critical Appraisal for Cross-sectional Studies Using the *Risk of Bias Instrument for Cross Sectional Surveys of Attitudes and Practices* Tool (n=7)

| **Study** | **Is the response rate adequate?** | **Is the source population representative of the population of interest?** | **Is the survey clinically sensible?** | **Is there any evidence for the reliability and validity of the survey instrument?** | **Is there little missing data?** | **Total Quality Assessment Score** |
| --- | --- | --- | --- | --- | --- | --- |
| **Oksanen (2022)** | Low | Low | Low | Low | Low | Low |
| **Gosse (2021)** | Unclear | Unclear | Low | High | Unclear | High |
| **Vargas (2021)** | High | High | Low | Low | Unclear | Moderate |
| **Vargas (2020)** | High | High | Low | Low | Unclear | Moderate |
| **Muhonen (2016)** | Low | Low | Unclear | Unclear | Unclear | Moderate |
| **Moutier (2016)** | High | High | Low | Unclear | Unclear | High |
| **Conco (2021)** | High | Low | Unclear | High | Unclear | High |

**The Risk of Bias Instrument for Cross-sectional Surveys of Attitudes and Practices:** All domains were categorized as having a low, moderate, or high risk of bias. Specifically, a low-risk bias response is “Low,” and a high-risk bias response can be either “High” or “Unclear”. Studies will be classified as having a low risk of bias if they score low risk on at least four domains. A study will be categorized as having a high risk of bias when at least four domains are high. Any score in between will be classified as a moderate risk of bias.

**Supplementary Table 2:** Critical Appraisal for qualitative studies using the *Critical Appraisal of Qualitative Studies* Tool (n=5)

| **Study** | **Was the qualitative approach appropriate?** | **Was the sampling strategy appropriate for the approach?** | **What were the data collection methods?** | **How were data analyzed and how were these checked?** | **Is the researcher’s position described?** | **Do the results make sense?** | **Are the conclusions drawn justified by the results?** | **Are the finding transferable to other clinical settings?** | **Total quality assessment score** |
| --- | --- | --- | --- | --- | --- | --- | --- | --- | --- |
| **Gorska et al. (2020)** | Yes | Yes | Yes | Unclear | No | Yes | Yes | Unclear | Moderate |
| **Lipton et al. (2020)** | Unclear | Unclear | Unclear | Unclear | Unclear | Yes | Yes | Unclear | High |
| **Mawere et al. (2022)** | Yes | Yes | Yes | Yes | Unclear | Yes | Yes | No | Low |
| **Oleto et al. (2022)** | Yes | Yes | Yes | Yes | Unclear | Yes | Yes | No | Low |
| **Sougou et al. (2022)** | Yes | Unclear | Yes | Yes | Unclear | Yes | Yes | Unclear | Moderate |

**The Risk of Bias Instrument for Critical Appraisal of Qualitative Studies:** All domains were categorized as having a low, moderate or high risk of bias. Low risk of bias response options are “Yes,” and high risk of bias responses are “No” or “Unclear.” Low risk studies are classified if low risk of bias is scored on at least 6 domains, high risk of bias was categorized when at least 6 domains were high, and anything in between is classified as moderate risk of bias.

**Supplementary Table 3:** Critical appraisal for mixed methods studies using the *Mixed methods Appraisal Tool (MMAT)* (n=4)

| **Study** | **Is there an adequate rationale for using a mixed methods design to address the research question?** | **Are the different components of the study effectively integrated to answer the research question?** | **Are the outputs of the integration of qualitative and quantitative components adequately interpreted?** | **Are divergences and inconsistencies between quantitative and qualitative results adequately addressed?** | **Do the different components of the study adhere to the quality criteria of each tradition of the methods involved?** | **Total quality assessment score** |
| --- | --- | --- | --- | --- | --- | --- |
| **Banner et al. (2022** | No | Yes | Yes | Yes | Yes | *#### |
| **Bar-walker et al. (2021)** | No | Yes | Yes | Yes | Yes | #### |
| **Berlingo et al. (2018)** | No | Yes | Yes | Yes | Yes | #### |
| **Martinez et al. (2017)** | No | No | No | Yes | Yes | **### |

**The Risk of Bias Instrument for Mixed Methods Studies:** All domains are categorized as having, low, moderate or high risk of bias. Low risk of bias responses are “Yes” and high risk of bias responses are “No”. If a score of 60% or higher is achieved, the study is considered to have a low risk of bias and if a study receives a score of 40% or lower, it is considered to have a high risk of bias.

*#### indicates a score of 80%

**### indicates a score of 60%

**Supplementary Table 4**: Recommendations for Practice, Policy, and Research

| **Practice** | - Educate and provide training to all positions within academic hierarchies (e.g. chair, faculty, Human Resource Professionals, course coordinators) regarding the rising concerns of GBV within virtual and physical academic workspaces. - Continue to provide a safe and confidential environment for reporting GBV with timely follow up and appropriate investigations. - Consider external review of GBV incidents with a panel of individuals that are not associated with the organizational structure of the institution.   - Provide mental health and social resources support to those who report GBV, to empower them and protect others in the workplace.   - Reporting program, hybrid mode. Give learners and faculty the option of choosing between the anonymous reporting platform or going in person to a confidential office to report. |
| --- | --- |
| **Policy** | - Revise and implement training courses/sessions on workplace psychological safety. - Broaden workplace policies on GBV to promote inclusivity and provide justice to those historically marginalized in masculinized work environments. - Involve stakeholders (learners, faculty, leadership and senior management) from different intersectional groups and those with lived experience of GBV in academic workspaces to ensure that the anti-GBV policies are relevant and effective to address and re-dress GBV. |
| **Research** | - Continue to investigate gender identity disparities using an intersectional approach to improve reporting the prevalence rate and research recommendations to combat GBV in academic workspaces. - Further research on GBV interventions and practices. |

**Supplementary Figure 3 Funnel Pots**

| 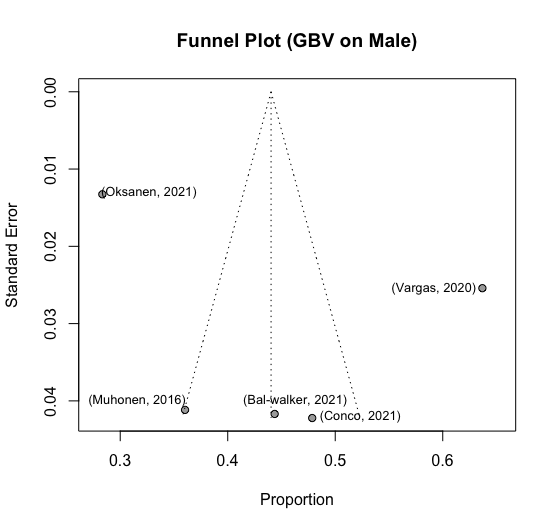  **3a. Funnel plot for Gender-Based Violence, Males:** The funnel plot suggests potential publication bias or some form of underlying systematic issues within the literature. This indicates to viewers that smaller studies that displayed smaller effects are not publishing their results. | 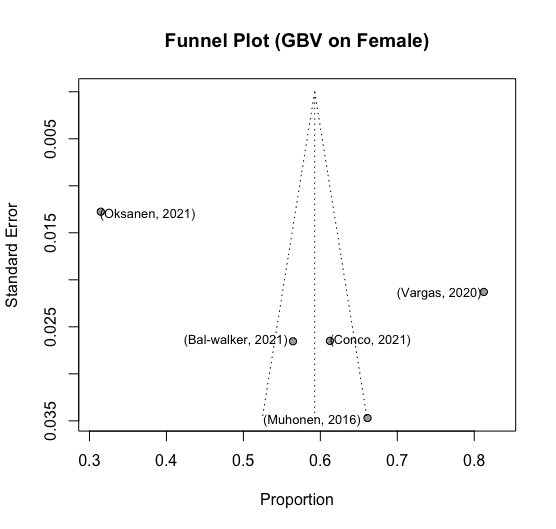  **3b. Funnel Plot for Gender-Based Violence, Females:** This funnel plot suggests a potential bias due to the asymmetries in the distribution of the data points. This could indicate that smaller studies that show of an effect are not being published in the literature. | |
| --- | --- | --- |
| 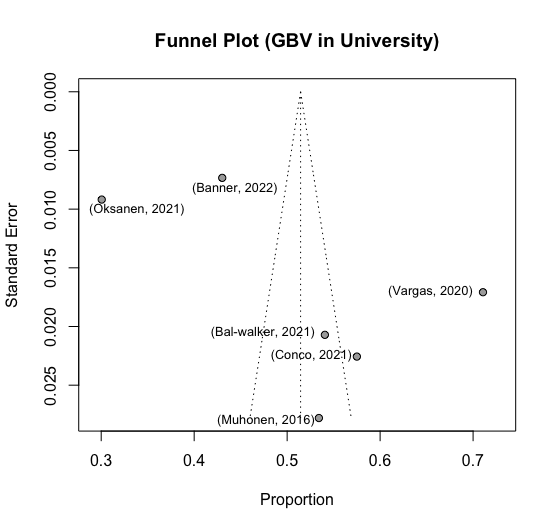  **3c. Funnel Plot for Gender-Based Violence, University:** The asymmetry in this plot suggests that there is publication bias or some other underlying systemic issue within the research. | 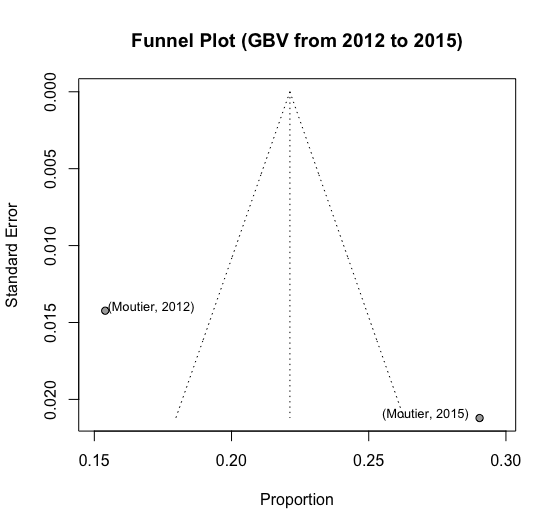  **3d. Funnel Plot for Gender-Based Violence, 2012 to 2015:** Since this plot only has two points of data, it is difficult to assess the presence of bias. Both points, however, seem to be located symmetrically around the vertical line, suggesting a lack of bias. |  |
