# Supplementary material for: Examining the Prevalence and Effects of Gender-based Violence in Academic Settings: A Systematic Review and Meta-analyses
Source: Trauma Violence Abuse. 2024 Oct 23;26(4):755–68. doi: 10.1177/15248380241289436 (PMC12397552; doi:10.1177/15248380241289436)
Supplement: sj-docx-2-tva-10.1177_15248380241289436 – Supplemental material for Examining the Prevalence and Effects of Gender-based Violence in Academic Settings: A Systematic Review and Meta-analyses [file sj-docx-2-tva-10.1177_15248380241289436.docx]

**Appendix A**

**Databases and Search Results**

**CINAHL – 305 articles**

1. (Gender-based violence or GBV or Harassing or Harassment or Cyberbullying or Cyberhate or Cyberharassment or Gender discrimination or violence victim or exploitation or bullying or intimidation)
2. ((Virtual or web or Online or Remote or Digital or Internet or telecommuting or teleworking) ) AND ( (work or employment or labour or workplace))
3. (university or academia or college or healthcare or Skilled trades or industry)
4. S1 AND S2
5. S3 AND S4

**OVID – 361 articles**

1. (Gender-based violence or GBV or Harassing or Harassment or Cyberbullying or Cyberhate or Cyberharassment or Gender discrimination or violence victim or exploitation or bullying or intimidation).tw,kf.
2. ((Virtual or web or Online or Remote or Digital or Internet or telecommuting or teleworking) adj2 (work or employment or labour or workplace)).tw,kf.
3. (university or academia or college or healthcare or Skilled trades or industry).tw,kf.
4. 1 and 2
5. 3 and 4

**PubMed – 122 articles**

("university"[Title/Abstract] OR "academia"[Title/Abstract] OR "college"[Title/Abstract] OR "healthcare"[Title/Abstract] OR "skilled trades"[Title/Abstract] OR "industry"[Title/Abstract]) AND ("gender based violence"[Title/Abstract] OR "GBV"[Title/Abstract] OR "Harassing"[Title/Abstract] OR "Harassment"[Title/Abstract] OR "Cyberbullying"[Title/Abstract] OR "Cyberhate"[Title/Abstract] OR "Cyberharassment"[Title/Abstract] OR "gender discrimination"[Title/Abstract] OR "violence victim"[Title/Abstract] OR "exploitation"[Title/Abstract] OR "bullying"[Title/Abstract] OR "intimidation"[Title/Abstract]) AND (("Virtual"[Title/Abstract] OR "web"[Title/Abstract] OR "Online"[Title/Abstract] OR "Remote"[Title/Abstract] OR "Digital"[Title/Abstract] OR "Internet"[Title/Abstract] OR "telecommuting"[Title/Abstract] OR "teleworking"[Title/Abstract]) AND ("work"[Title/Abstract] OR "employment"[Title/Abstract] OR "labour"[Title/Abstract] OR "workplace"[Title/Abstract]))

**Scopus - 57 articles**

((TITLE-ABS-KEY("gender-based violence"  OR  gbv  OR  harassing  OR  harassment  OR  cyberbullying  OR  cyberhate  OR  cyberharassment  OR  "gender discrimination"  OR  "violence victim"  OR  exploitation  OR  bullying  OR  intimidation ) )  AND  ( TITLE-ABS-KEY ( virtual  OR  web  OR  online  OR  remote  OR  digital  OR  internet  OR  telecommuting  OR  teleworking  W/2  work  OR  employment  OR  labour  OR  workplace ) ) )  AND  ( TITLE-ABS-KEY ( university  OR  academia  OR  college  OR  healthcare  OR  "Skilled trades"  OR  industry ) )

AND  ( LIMIT-TO ( PUBYEAR ,  2023 )  OR  LIMIT-TO ( PUBYEAR ,  2022 )  OR  LIMIT-TO ( PUBYEAR ,  2021 )  OR  LIMIT-TO ( PUBYEAR ,  2020 )  OR  LIMIT-TO ( PUBYEAR ,  2019 )  OR  LIMIT-TO ( PUBYEAR ,  2018 )  OR  LIMIT-TO ( PUBYEAR ,  2017 )  OR  LIMIT-TO ( PUBYEAR ,  2016 )  OR  LIMIT-TO ( PUBYEAR ,  2015 )  OR  LIMIT-TO ( PUBYEAR ,  2014 )  OR  LIMIT-TO ( PUBYEAR ,  2013 ) )

**Web of science – 629 articles**

1. TS=(Gender-based violence or GBV or Harassing or Harassment or Cyberbullying or Cyberhate or Cyberharassment or Gender discrimination or violence victim or exploitation or bullying or intimidation)
2. TS=(Virtual or web or Online or Remote or Digital or Internet or telecommuting or teleworking) AND (work or employment or labour or workplace)
3. TS=(university or academia or college or healthcare or Skilled trades or industry)
4. #1 AND #2
5. #3 AND #4
